# Rice small protein OsS1Fa1 participates in stress responses as an inner nuclear membrane protein

**DOI:** 10.1080/15592324.2024.2439252

**Published:** 2024-12-09

**Authors:** Wang Ki Min, Kyu Ho Lee, Jong Tae Song, Hak Soo Seo

**Affiliations:** a Department of Agriculture, Forestry and Bioresources, Research Institute for Agriculture and Life Sciences, and Plant Genomics and Breeding Institute, Seoul National University, Seoul, Korea; b Department of Applied Biosciences, Kyungpook National University, Daegu, Korea; c Bio-MAX Institute, Seoul National University, Seoul, Korea

**Keywords:** Rice, OsS1Fa1, inner nuclear membrane, OsSUN1, defense, stress

## Abstract

The rice small protein OsS1Fa1, a homolog of spinach S1Fa, plays a significant role in drought tolerance, attributed to its transmembrane domain. In this study, we aim to further elucidate the potential roles of OsS1Fa1 in cold and biotic stresses as an inner nuclear membrane protein. Fluorescence analysis confirmed the localization of OsS1Fa1 to the inner nuclear membrane. Utilizing the bimolecular fluorescence complementation (BiFC) and bacterial infiltration assays with OsS1Fa1 and the inner nuclear membrane protein OsSUN1 (Rice Sad1 and UNC84 (SUN) domain containing 1 (SUN1)), we observed fluorescence detection within the inner nuclear membrane, indicating a direct interaction and colocalization between OsS1Fa1 and OsSUN1. Expression analysis revealed that overexpression of OsS1Fa1 induced the expression of various genes associated with cold and defense responses, including *COLD-REGULATED 15A* (*COR15A*), *PATHOGENESIS-RELATED PROTEIN 1* (*PR1*), and *PLANT DEFENSIN 1.2* (*PDF1.2*). Our findings collectively indicate that OsS1Fa1 plays crucial roles in both abiotic and biotic stress tolerance as an inner nuclear membrane protein.

## Results and discussion

Small peptides or proteins are composed of fewer than 100 amino acids in general. They play important roles in cell proliferation and root development,^1^ pollen fertility,^2^ stomata movement,^3^ nutrient absorption_1_, pest and disease resistance,^4,5^ and environment adaptation.^6^

S1Fa is a small peptide with 76 amino acids identified in spinach (*Spinacia oleracea* L.)^7^ and conserved in dicotyledonous and monocotyledonous plants.^8^ Rice S1Fa1, a homolog of spinach S1Fa, is also composed of 76 amino acids and contains four distinct motifs, a nuclear localization signal (NLS), a DNA recognition motif, a transmembrane domain, and a sumoylation motif.^9^

The *S1Fa* gene is expressed in seedlings, leaves, and roots during development,^8–10^ and overexpression of OsS1Fa1 in Arabidopsis enhances drought stress tolerance.^9^ In addition, the transmembrane domain of OsS1Fa1 is responsible for drought tolerance and determines its subcellular localization.^11^ Furthermore, OsS1Fa1 is modified with SUMO. Our findings indicate that the four different motifs play distinct roles in the function of OsS1Fa1 including drought tolerance and subcellular localization.

**Figure 1.**
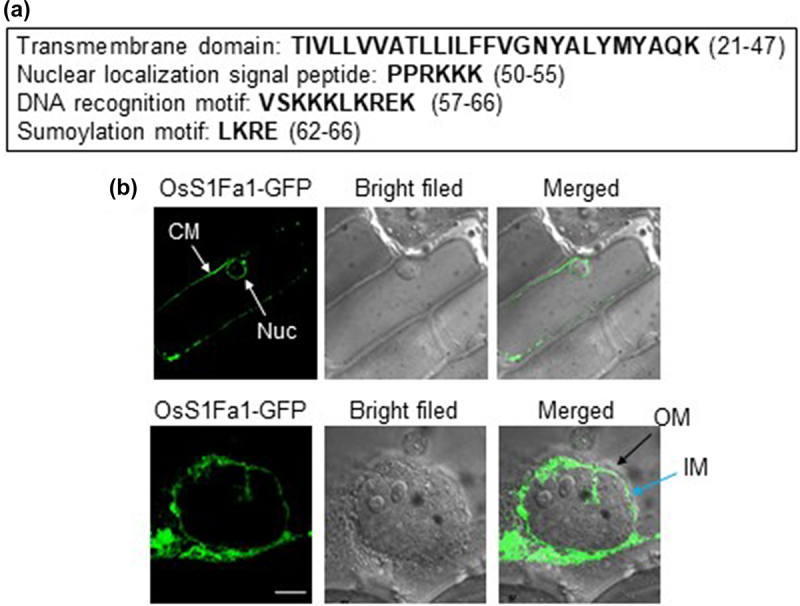
Analysis of the association of OsS1Fa1-gfp with the inner nuclear membrane in onion epidermal cells. (a) Amino acid sequences of each domain of OsS1Fa1 are shown. (b) The *35S-OsS1Fa1-gfp* construct was introduced into onion epidermal cells by particle bombardment and then the bombarded cells were incubated at 25°C in the dark for 24 h. GFP signal was visualized using CLSM. CM, cytoplasmic membrane; nuc, nucleus; IM, inner nuclear membrane; OM, outer nuclear membrane. Scale bar, 5 µm.

In this work, we extended our study to investigate subcellular localization in detail and elucidate the possible role of OsS1Fa1 in abiotic and biotic stresses. Since OsS1Fa1 contains four distinct motifs and is localized to both the cytoplasmic and nuclear membranes (Figure 1a, Ref 9), we further analyzed whether it localizes to the inner or outer nuclear membrane. To do this, we first generated a recombinant plasmid *35S-OsS1Fa1-GFP* that coding sequence of the *green fluorescence protein* (*GFP*) gene was cloned in-frame to the 3’ end of *OsS1Fa1* under the control of the cauliflower mosaic virus (CaMV) *35S* promoter. After introducing the *35S-OsS1Fa1-GFP* construct into onion epidermal cells by particle bombardment technique, we examined GFP fluorescence using confocal laser scanning microscopy (CLSM). The GFP fluorescence appeared to be localized to the inner nuclear membrane (Figure 1b).

**Figure 2.**
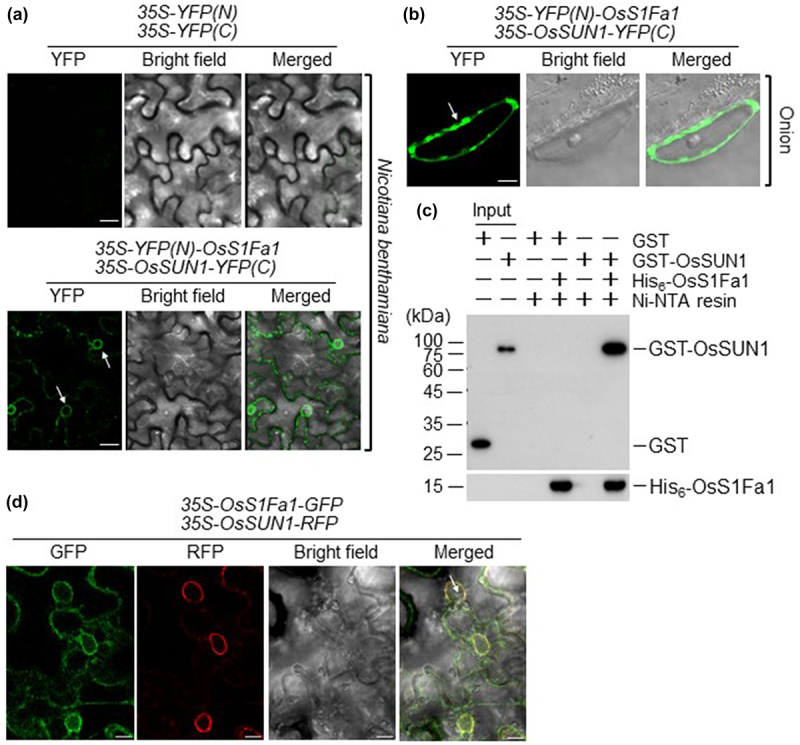
OsS1Fa1 interacts with OsSUN1. The interaction between OsS1Fa1 and OsSUN1 was examined using the BiFC assay. Two sets of constructs, 35S-YFPN and 35S-YFPC, as well as 35S-YFPN-OsS1Fa1 and 35S-OsSUN1-yfpc, were introduced into *Nicotiana benthamiana* leaves by bacterial infiltration (a) or onion epidermal cells by particle bombardment (b), and fluorescence signals were detected using CLSM. Scale bar, 10 µm (a) or 2 µm (b). White arrows indicate the nucleus. (c) The interaction between OsS1Fa1 and OsSUN1 was further examined using an *in vitro* pull-down assay. GST, GST-OsSUN1, and His_6_-OsS1Fa1 proteins were overexpressed in *E. coli* and purified using glutathione or Ni^2+^-NTA affinity columns. The GST-OsSUN1 protein was pulled down with the His_6_-OsS1Fa1 protein, separated on 11% sds-polyacrylamide gels, and analyzed by western blotting using anti-gst antibody. The presence of His_6_-OsS1Fa1 protein was detected by western blotting using anti-His antibody. (d) Subcellular localization of OsS1Fa1 and OsSUN1. Inner nuclear membrane localization of OsS1Fa1 and OsSUN1 was investigated via bacterial infiltration. Constructs harboring *35S-OsS1Fa1-gfp* and *35S-OsSUN1-rfp* were introduced into *N. benthamiana* leaves by bacterial infiltration. The bombarded tissues were analyzed by CLSM to visualize transient GFP or RFP expression. DIC, differential interference contrast. Bar, 10 μm.

Next, to confirm the localization of OsS1Fa1 to the inner nuclear membranes and speculate on its potential role as an inner nuclear membrane protein, we investigated the interaction between OsS1Fa1 and OsSUN1. OsSUN1 is a rice SUN domain protein known to function as a component of the inner nuclear membrane (INM).^12^ To accomplish this, we utilized bimolecular fluorescence complementation (BiFC) and *in vitro* pull-down assays. For BiFC assay, cDNAs of *OsS1Fa1* and *OsSUN1* were cloned into the Gateway vectors: pSAT4-DEST-n(174)EYFP-C1 or pSAT5-DEST-c(175-end)EYFP-C1. The fusion constructs, encoding EYFPN-OsS1Fa1 and OsSUN1-EYFPC proteins, were introduced into *Agrobacterium tumefaciens*, and the transformed cells were co-injected into *Nicotiana benthamiana* leaves. YFP signal was detected in infiltrated leaves using CLSM. The results showed that fluorescence was observed in the nuclear membrane (Figure 2a), indicating a direct interaction between OsS1Fa1 and OsSUN1 *in vivo* and the localization of OsS1Fa1 to the inner nuclear membrane. We also confirmed the direct interaction between OsS1Fa1 and OsSUN1 and their localization in the inner nuclear membrane in onion epidermal cells as well (Figure 2b). For the *in vitro* pull-down assay, we produced recombinant proteins, His_6_-OsS1Fa1 and glutathione S-transferase (GST)-OsSUN1. To produce His_6_-OsS1Fa1 and GST-OsSUN1, cDNAs encoding full-length OsS1Fa1 and OsSUN1 were cloned into the pET28a and pGEX4T–1 vectors, respectively. Recombinant proteins His_6_-OsS1Fa1 and GST-OsSUN1 were overexpressed in *E. coli* and then purified using Ni^2+^-nitrilotriacetate (NTA) and glutathione resins, respectively. Then, the purified GST-OsSUN1 protein was subsequently incubated with OsS1Fa1 and pulled down with Ni^2+^-NTA resin. The bound GST-OsSUN1 protein was separated by 11% SDS-PAGE and analyzed by western blotting with anti-GST antibody. The results demonstrated that OsS1Fa1 effectively pulled down GST-OsSUN1, but not GST (Figure 2c), providing further evidence of the interaction between OsS1Fa1 and OsSUN1. Moreover, we analyzed the subcellular localization patterns of OsS1Fa1 and OsSUN1 in more detail. The *35S-OsS1Fa1-GFP* and *35S-OsSUN1-RFP* constructs were agroinfiltrated into *N. benthamiana* leaves, and GFP or RFP signals were detected using CLSM. Figure 2d shows that OsS1Fa1-GFP and OsSUN1-RFP are localized in the inner nuclear membrane, although OsS1Fa1-YFP was also found in cytoplasmic regions that did not contain OsSUN1, which was strictly localized to the inner nuclear membrane. Taken together, these findings indicate that OsS1Fa1 and OsSUN1 co-localize within the inner nuclear membrane through a direct interaction *in vivo*.

In animal systems, it is well known that SUN1 plays a major role in nuclear-cytoplasmic connection by formation of a bridge across the nuclear envelope, known as the LINC (Linker of nucleoskeleton and cytoskeleton) complex, via interaction with the conserved luminal KASH (Klarsicht/ANC-1/Syne-1 homology) domain of nesprins located in the outer nuclear membrane (ONM).^13^ A number of studies have shown that loss of LINC complexes reduces nuclear and cellular rigidity, increasing tissue fluidity, promoting invasive activity, and inducing cancer progression.^14–17^ Additionally, studies have uncovered a fundamental role of SUN1 in nuclear structure determination function,^17^ suggesting that the effects of SUN1 on regulating nuclear architecture affect biological function in cancer cells. Abnormal expression of SUN2 and LINC complexes is associated with the occurrence of many human diseases, especially cancers,^14^ indicating that dysregulation of SUN protein is related to cancer development. Furthermore, SUN1 involves in the resistance to DNA damage and maintaining the genome integrity.^18^ In plants, it known that Arabidopsis AtSUN1, rice OsSUN1, and maize ZmSUN2 involve in meiosis.^12,19,20^ Together with our findings, these observations suggest that OsS1Fa1 participates in various cellular processes by regulating the function of OsSUN1.

**Figure 3.**
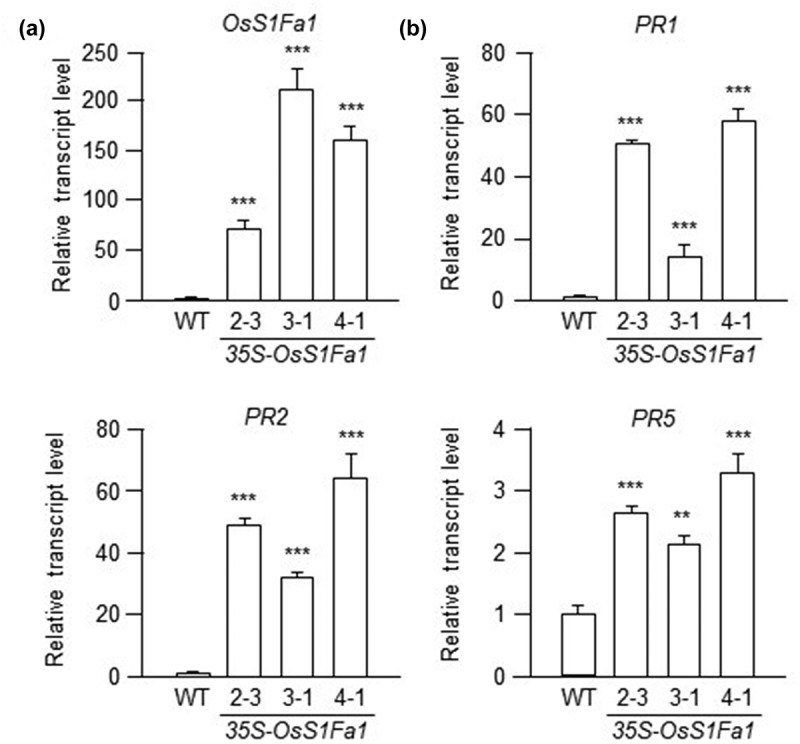
Expression of SA signaling-related genes in transgenic Arabidopsis lines overexpressing OsS1Fa1. (a) Total RNAs were isolated from WT and three *OsS1fa*-overexpressing transgenic plants and the levels of *OsS1fa1* transcripts were examined by qRT-pcr with *OsS1fa1*-specific primers. Data represent mean ± SD (*n* = 3). Asterisks (***) indicate statistically significant differences in transcript levels (*p* < 0.001; Student’s *t*-test) between WT and indicated *OsS1fa1*-overexpressing transgenic Arabidopsis lines. (b) Expression analysis of *PR*1, *PR*2, and *PR5* genes in WT and transgenic plants by rt-qPCR using gene-specific primers. Total RNA samples used in this experiment were the same as those used in (a). Data represent mean ± SD (*n* = 3). Asterisks indicate statistically significant differences in transcript levels (**p < 0.01; ***p < 0.001; Student’s *t*-test) between WT and indicated *OsS1fa1*-overexpressing transgenic Arabidopsis lines.

**Figure 4.**
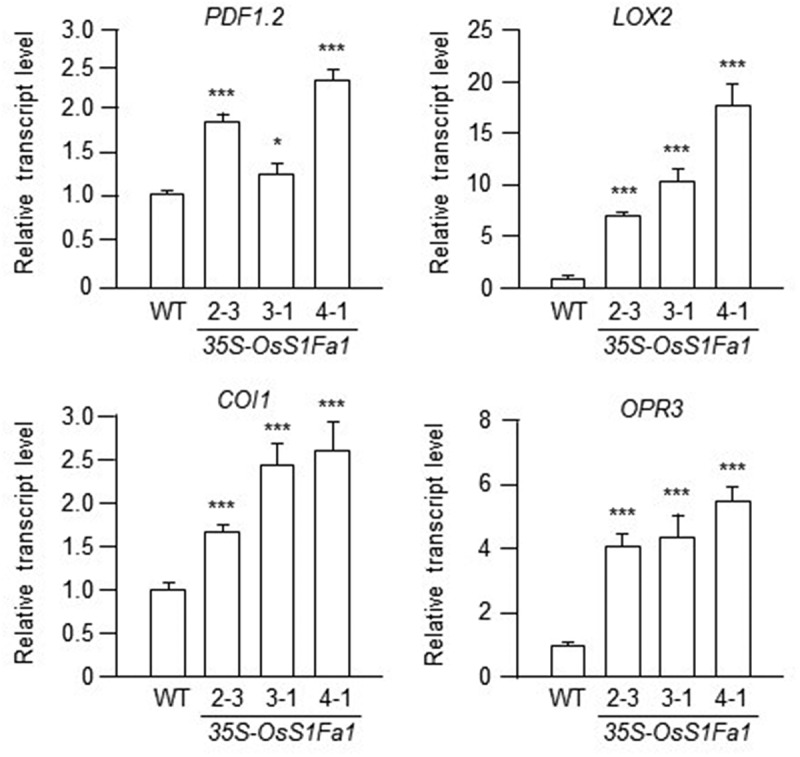
Expression of JA signaling-related genes in transgenic Arabidopsis lines overexpressing OsS1Fa1. Expression analysis of *PDF1.2*, *LOX2*, *COI1* and *OPR3* genes in WT and transgenic plants by rt-qPCR using gene-specific primers. Total RNA samples used in this experiment were the same as those used in Figure 3a. Data represent mean ± SD (*n* = 3). Asterisks indicate statistically significant differences in transcript levels (*p < 0.05; ***p < 0.001; Student’s *t*-test) between WT and indicated *OsS1fa1*-overexpressing transgenic Arabidopsis lines.

**Figure 5.**
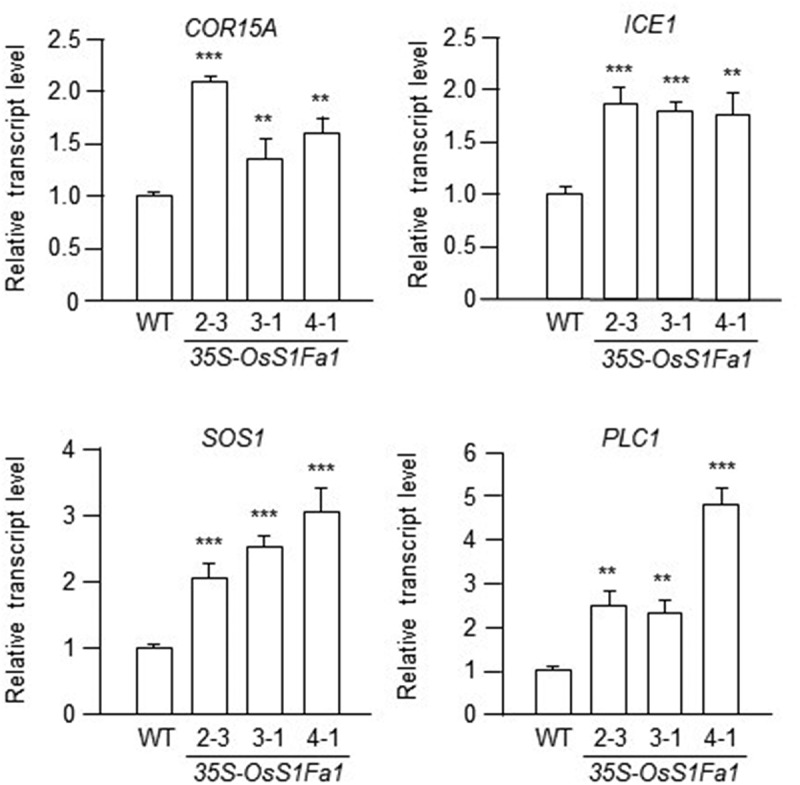
Expression of cold stress-related genes in transgenic Arabidopsis lines overexpressing OsS1Fa1. Expression analysis of *COR15A*, *ICE1*, *SOS1* and *PLC1* genes in WT and transgenic plants by rt-qPCR using gene-specific primers. Total RNA samples used in this experiment were the same as those used in Figure 3a. Data represent mean ± SD (*n* = 3). Asterisks indicate statistically significant differences in transcript levels (**p < 0.01; ***p < 0.001; Student’s *t*-test) between WT and indicated *OsS1fa1*-overexpressing transgenic Arabidopsis lines.

Since *OsS1Fa1* expression was upregulated under drought stress, along with increased expression of drought stress-related genes in OsS1Fa1-overexpressing transgenic Arabidopsis plants compared to wild-type (WT) plants,^11^ we expanded our investigation to explore the potential role of OsS1Fa1 under various abiotic and biotic stresses. To do so, we examined the expression of defense- and cold stress-related genes. In our previous study, we utilized three distinct transgenic Arabidopsis plants expressing varying levels of OsS1Fa1 proteins,^9^ which we continued to use in this study. Firstly, we analyzed the expression of salicylate signaling-related genes in both WT and OsS1Fa-overexpressing transgenic plants using real time-quantitative polymerase chain reaction (RT-qPCR). Total RNA was extracted from the leaves of WT and three distinct *OsS1Fa1*-overexpressing lines (Figure 3a) grown for 14 days in a growth chamber at 22°C under a 16-hour light/8-hour dark cycle on Murashige and Skoog (MS) medium. RT-qPCR was carried out using gene-specific primers. The results revealed elevated transcript levels of *PATHOGENESIS-RELATED PROTEIN 1* (*PR1*), *PATHOGENESIS-RELATED PROTEIN* 2 (*PR2*), and *PATHOGENESIS-RELATED PROTEIN 5* (*PR5*) in OsS1Fa-overexpressing transgenic plants compared to the wild type (Figure 3b). Next, we examined the expression of jasmonate signaling-related genes, *PLANT DEFENSIN 1.2* (*PDF1.2*), *LIPOXYGENASE 2* (*LOX2*), *CORONATINE INSENSITIVE1* (*COI1*), and *OXOPHYTODIENOATE-REDUCTASE 3* (*OPR3*), under the same conditions. The results indicated higher expression levels of these genes in OsS1Fa-overexpressing transgenic plants than in the wild type (Figure 4). Finally, we assessed the expression of cold stress-related genes, *COLD-REGULATED 15A* (*COR15A*), *INDUCER OF CBF/DREB1 EXPRESSION 1* (*ICE1*), *SALT OVERLY SENSITIVE 1* (*SOS1*), and *PHOSPHOINOSITIDE PHOSPHOLIPASE C1* (*PLC1*), under the same conditions. Similarly, their transcript levels were elevated in OsS1Fa-overexpressing transgenic plants compared to the wild type (Figure 5).

**Figure 6.**
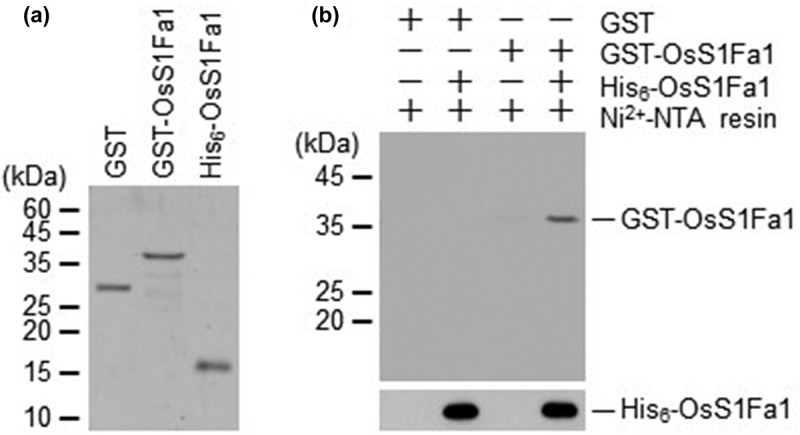
OsS1Fa forms homodimer. (a) GST, GST-His_6_-OsS1Fa1 and His_6_-OsS1Fa1 were overexpressed in *E. coli* and purified with glutathione or Ni^2+^-NTA affinity columns. (b) The GST-His_6_-OsS1Fa1 protein was pulled down with the His_6_-OsS1Fa1 protein, separated on 11% sds-polyacrylamide gels, and analyzed by western blotting with anti-gst antibody. His_6_-OsS1Fa1 protein was also detected by western blotting with anti-His antibody.

Many transcription factors function as a homodimer. We thus examined whether OsSf1Fa1 form a homodimer or not. To do this, we produced recombinant proteins, His_6_-OsS1Fa1 and GST-OsS1Fa1. cDNA encoding full-length OsS1Fa1 was cloned into the pET28a or the pGEX4T–1 vectors. Recombinant proteins His_6_-OsS1Fa1 and GST-OsS1Fa1 were overexpressed in *E. coli* and then purified using Ni^2+^-NTA and glutathione resins, respectively (Figure 6a). The purified proteins were incubated together and then pulled down with Ni^2+^-NTA resin. The bound GST-OsS1Fa1 protein was separated by 11% SDS-PAGE and analyzed by western blotting with anti-GST antibody. The results clearly showed that His_6_-OsS1Fa1 effectively pulled down GST-OsS1Fa1, but not GST (Figure 6b), indicating that OsS1Fa1 forms homodimer and suggesting that it can function as a homodimer.

The change in nuclear shape reflects the changes in chromatin structure to modulate gene accessibility. For an instance, NaCl-induced hyperosmotic stress was shown to disrupt chromatin organization, with associated transcriptional changes.^21^ The change in nuclear shape reflects the changes in differences in nuclear lamina composition resulting in altered nuclear stiffness. For example, hyperosmotic stress decreases nuclear circularity and size and increases nuclear stiffness in meristematic cells.^22^ These indicate that nuclear architecture, and nuclear and cellular rigidity can be involved various stress responses. By the way, SUN proteins also participate in nuclear architecture, and nuclear and cellular rigidity,^19,23^ suggesting that it plays an important role in the abiotic stress tolerance and disease resistance by modulating and maintaining cellular shape such as nucleus and cytoplasm structures. Because OsS1Fa1 directly interacted with OsSUN1 in INM (Figure 2) and plays a role in drought tolerance,^9,11^ we speculated that OsS1Fa1 must be involved in various stresses. Our investigation revealed that the expression of cold stress-related genes and SA and JA signaling-related genes was upregulated in OsS1Fa1-overexpressing Arabidopsis plants (Figures 3–5). SA-related genes are involved in the resistance to the diseases by virus and bacteria,^24^ and JA-related genes are involved in the resistance to the diseases by fungi and insects and to physical damages.^25^ Therefore, our findings indicate that OsS1Fa1 involves in various abiotic stress tolerance and disease resistance alone or as a complex with OsSUN1.
